# Evaluation of Cardiac Function Index as Measured by Transpulmonary Thermodilution as an Indicator of Left Ventricular Ejection Fraction in Cardiogenic Shock

**DOI:** 10.1155/2014/598029

**Published:** 2014-06-11

**Authors:** Jessica Perny, Antoine Kimmoun, Pierre Perez, Bruno Levy

**Affiliations:** ^1^CHU Nancy, Service de Reanimation Medicale Brabois, Pole Cardiovasculaire et Reanimation Medicale, Hopital Brabois, 54511 Vandoeuvre-les-Nancy, France; ^2^INSERM, CHU Nancy, Groupe Choc Inserm, U1116, Faculté de Médecine, 54511 Vandoeuvre-les-Nancy, France; ^3^Université de Lorraine, 54000 Nancy, France

## Abstract

*Introduction*. The PiCCO transpulmonary thermodilution technique provides two indices of cardiac systolic function, the cardiac function index (CFI) and the global ejection fraction (GEF). Both appear to be correlated with left ventricular ejection fraction (LVEF) measured by echocardiography in patients with circulatory failure, especially in septic shock. The aim of the present study was to test the reliability of CFI as an indicator of LVEF in patients with cardiogenic shock. *Methods*. In thirty-five patients with cardiogenic shock, we performed (i) simultaneous measurements of echocardiography LVEF and cardiac function index assessed by transpulmonary thermodilution (*n* = 72) and (ii) transpulmonary thermodilution before/after increasing inotropic agents (*n* = 18). *Results*. Mean LVEF was 31% (+/−11.7), CFI 3/min (+/−1), and GEF 14.2% (+/−6). CFI and GEF were both positively correlated with LVEF (*P* < 0.0001, *r* ^2^ = 0.27). CFI and GEF were significantly increased with inotropic infusion (resp., *P* = 0.005, *P* = 0.007). A cardiac function index <3.47/min predicted a left ventricular ejection fraction ≤35% (sensitivity 81.1% and specificity 63%). In patients with right ventricular dysfunction, CFI was not correlated with LVEF. *Conclusion*. CFI is correlated with LVEF provided that patient does not present severe right ventricular dysfunction. Thus, the PiCCO transpulmonary thermodilution technique is useful for the monitoring of inotropic therapy during cardiogenic shock.

## 1. Introduction

Hemodynamic monitoring is essential for the diagnosis and therapeutic management of critically ill patients [1]. There are several different methods and techniques for monitoring patients with circulatory failure, although none are ideal (namely non-invasive, safe, reproducible, assessing cardiac preload and myocardial function) [2–5]. Considering the systolic function of the left ventricle, Doppler echocardiography has become the standard tool for measuring left ventricular ejection fraction (LVEF) [6, 7]. Unfortunately, echocardiography for hemodynamic monitoring is limited by the availability of equipment and/or experienced examiners on a 24/24 hour basis. As a result, the PiCCO system (Pulsion Medical System, Munich, Germany) based on the use of a specific thermodilution arterial catheter and a central venous line has emerged as an interesting monitoring approach and could be proposed as an alternative to echocardiography for the estimation of LVEF; indeed, this system allows on the one hand the assessment of cardiac output [5], of cardiac preload [3, 8–13], and on the other hand two indices of cardiac systolic function, the cardiac function index (CFI) and the global ejection fraction (GEF). Both appear to be correlated with left ventricular systolic ejection fraction measured with echocardiography in patients with circulatory failure [14–16]. However, these latter studies essentially pertained to patients with septic shock, and less than 15% of the patients presented severe heart failure (cardiogenic shock).

The aim of the present study was thus to evaluate the reliability of CFI as a marker of left ventricular ejection fraction in patients with cardiogenic shock. We hypothesized that CFI is correlated with LVEF, increases with inotropic infusion and is not altered with fluid expansion in patients with cardiogenic shock.

## 2. Material and Methods

### 2.1. Study Population

This prospective observational study was conducted in a 13-bed ICU in a university hospital.

Patients were included if they met the following criteria: presence of a cardiogenic shock and monitoring by a transpulmonary thermodilution device. Inclusion was possible during the initial evolution of cardiogenic shock (i.e., when inotropic agent was introduced and/or when dobutamine posology was increased).

Cardiogenic shock was defined as a persistent hypotension resulting from heart failure in the presence of adequate intravascular volume [17]. Circulatory shock was diagnosed by observing hypotension, tachycardia, poor tissue perfusion such as oliguria, cool skin, mottled extremities, and cerebral hypoperfusion. Associated hemodynamic criteria included the following:

- persistent hypotension (systolic blood pressure ≤90 mm Hg or decrease in systolic arterial pressure >30% in known hypertensive patients),
- cardiac index (CI) less than 2.2 L/min per m² without dobutamine infusion and/or patient already receiving inotropic agent because of a low CI. CI was assessed either by echocardiography or by transpulmonary thermodilution [17, 18].

Exclusion criteria included the following: age <18 years; no available echocardiography (absence of sufficient echogenicity); septic shock and/or septic cardiomyopathy; and patient treated with intra-aortic balloon pumping (given that the thermodilution technique requires interrupting the treatment and real time arterial pulse contour analysis is not possible). Nonsinus rhythm (atrial fibrillation), right ventricular failure, and therapeutic hypothermia for cardiac arrest were not considered as exclusion criteria.

The following data were recorded: age, sex, past medical history (such as chronic cardiac insufficiency and nonsinus rhythm), simplified acute physiology score (SAPS II), cause of cardiogenic shock, need for mechanical support, renal replacement therapy, and use of vasopressor and/or inotropic agent during measurement and during the ICU stay.

### 2.2. Transpulmonary Thermodilution and Calculation of CFI and GEF

A 5-French thermistor-tipped catheter was placed into the femoral artery and a central venous catheter was inserted into a central vein (jugular or subclavian vein); both were connected to the PiCCO system. Cardiac output (CO) and volumetric parameters were measured with the thermodilution technique and obtained after injection of 15 mL of cold isotonic saline 0.9% (<8°C) via the central venous catheter.

The CO was calculated from the thermodilution curves, according to the Stewart-Hamilton algorithm; the mean of three consecutive injections was recorded.

Volumetric parameters were calculated from the mean transit time (MTt) and the exponential downslope time (DSt) of the thermodilution curve: (i) intrathoracic thermal volume (ITTV) was obtained by the product of CO × MTt; (ii) pulmonary thermal volume (PTV) was obtained by the product of CO × DSt; (iii) the global end-diastolic volume (GEDV) represented the difference between ITTV and PTV: GEDV = ITTV − PTV = CO * MTt – CO * DSt.

The PiCCO monitor automatically calculated the two cardiac systolic function indices, namely, CFI and GEF.

- CFI is the ratio between CO and GEDV: CFI = CO/GEDV, expressed in min^−1^.
- GEF is defined as the ratio of the stroke volume (SV) to the quarter of the GEDV: GEF = SV/(GEDV/4), expressed as a percentage.

The transpulmonary thermodilution parameters were recorded in 3 different situations:

1. each time an echocardiography was performed,
2. before and 30 minutes after the initiation or increase in inotropic agent,
3. before and immediately after a volume loading: the reason for administering fluid infusion was systematically recorded (response to a passive leg-raising, presence of respiratory changes in pulse pressure).

### 2.3. Echocardiography

A transthoracic echocardiography was performed with a Vivid 3 (Philips) by a specially trained cardiologist. LVEF was obtained by the biplane Simpson's method or was visually estimated [19, 20].

The operator systematically looked for evidence of a right ventricular dysfunction; a severe right heart failure was defined in this study by the association of three abnormalities [21, 22]:

1. visually right ventricular dilatation and/or right ventricular wall motion abnormalities,
2. tricuspid annular plane systolic excursion (TAPSE) ≤15 mm,
3. systolic pulmonary artery pressure (PAPs) ≥35 mmHg.

### 2.4. Statistical Analysis

Statistical analyses were performed using the GraphPad software, version 4.0, and STATA software, version 9.0.

Continuous variables are expressed as mean (+/− standard derivation). Categorical variables are expressed as percentages. The correlations were tested using a Pearson test.

The comparison of variables between before and after therapeutic intervention was performed with a nonparametric test (Wilcoxon matched pairs signed-rank test).

The receiver operating characteristic (ROC) curve was constructed to study the ability of CFI to predict a LVEF ≤35%.

A *P* < 0.05 was considered statistically significant.

## 3. Results

Thirty-five patients were studied between January 2009 and November 2012.  Table 1 summarizes the characteristics of the population. Of the 19 men (54.3%) and 16 women (45.7%), 21 patients (60%) had a preexisting cardiomyopathy, with a mean LVEF of 39.7% (+/−14), and 11 patients (31.5%) had a past history of chronic atrial fibrillation. Mean SAPS II score at admission was 54 (+/−21). The causes of cardiogenic shock were principally acute myocardial infarction (*n* = 15) and end-stage cardiomyopathy (*n* = 6). Overall ICU mortality rate was 54%.

**Figure 1:**
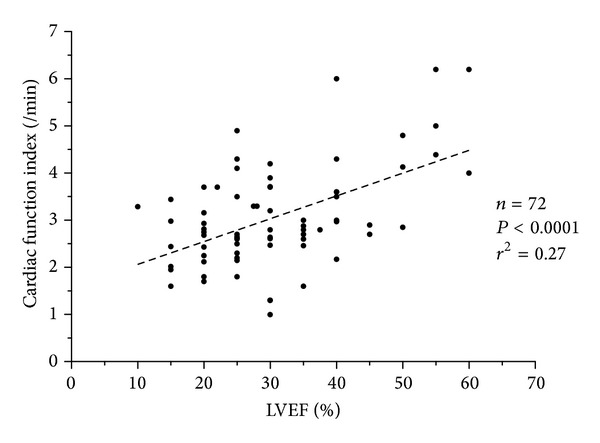
Correlation between cardiac function index (CFI) and left ventricular ejection fraction (LVEF). Dashed line: linear regression line.

Seventy-two pairs of CFI/LVEF measurements were obtained, for which thermodilution and echocardiography variables are described in Table 2. In 38 pairs, patients were treated with norepinephrine (mean dosage of 0.72 *μ*g/kg/min), and 63 were under dobutamine infusion (mean dosage of 9.4 *μ*g/kg/min). For the 72 pairs of measurements, mean LVEF was 31% (+/−11.7), whereas mean CI, CFI, and GEF were, respectively, 2.6 L/min/m^2^ (+/−0.8), 3/min (+/−1), and 14.2 (+/−6). As shown in Figure 1, a significant correlation between CFI and LVEF was observed (*P* < 0.0001, *r* = 0.52 (95% confidence interval: 0.32–0.67), *r* ^2^ = 0.27) in the 72 pairs of measurements. A significant correlation was also established between GEF and LVEF (*P* < 0.0001, *r* = 0.52 (95% confidence interval: 0.33–0.67), *r* ^2^ = 0.27).

**Figure 2:**
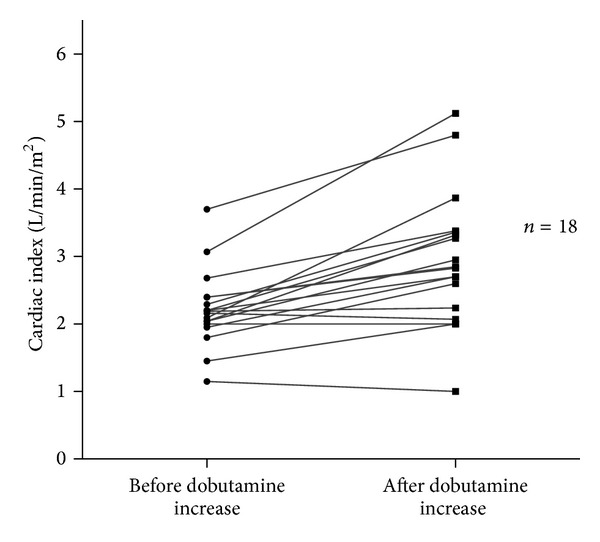
Changes in cardiac index with dobutamine infusion (*n* = 18): *P* = 0.0008.

**Figure 3:**
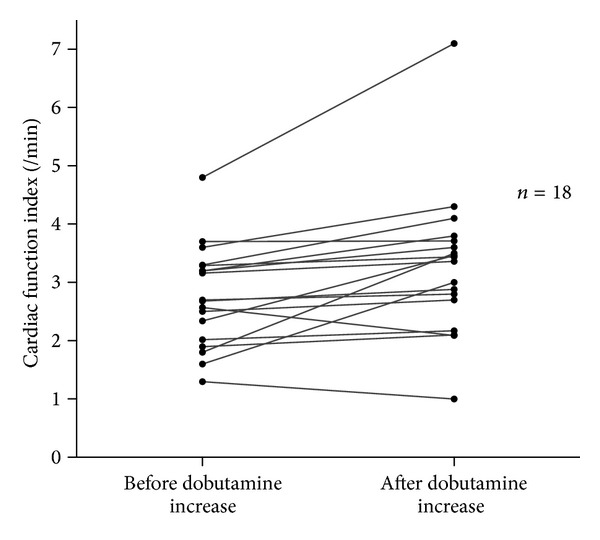
Changes in cardiac function index with dobutamine infusion (*n* = 18): increase in CFI under dobutamine (*P* = 0.0046).

Eighteen thermodilution measurements were obtained before and after the onset or increase in dobutamine infusion (Table 3). CI, CFI, and GEF were significantly increased with inotropic infusion, whereas there was no change in heart rate and GEDV (Table 3 and Figures 2 and 3).

Only 4 measurements before and after volume expansion were performed: 3 fluid loadings were decided because the patient was deemed to respond positively to fluid administration (presence of respiratory variation in pulse pressure in one patient; positive hemodynamic response to passive leg-raising in 2 others). CI, CFI, GEF, and GEDV were not altered by volume expansion (Table 4).

**Figure 4:**
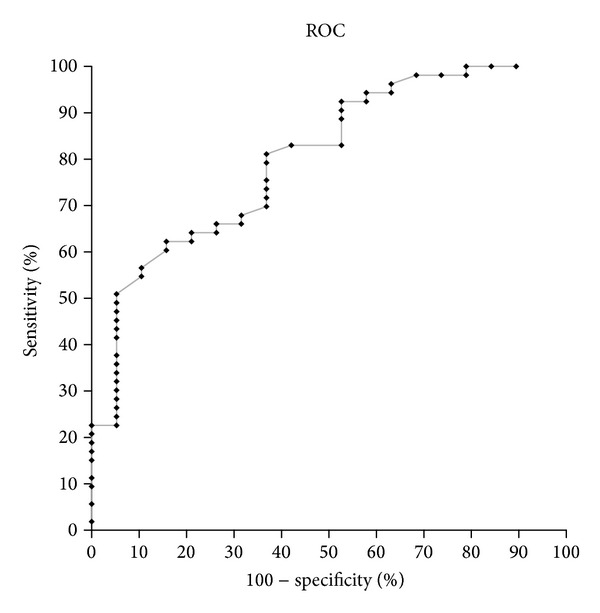
Receiver operating characteristic curve showing the ability of CFI to predict a LVEF ≤ 35%.

In the 72 pairs of measurements, a CFI value <3.47 allowed diagnosing a LVEF ≤35% with a sensitivity of 81.1% (95% confidence interval: 0.68–0.9) and a specificity of 63% (95% confidence interval: 0.38–0.83). The area under the ROC curve was 0.8 (95% confidence interval: 0.69–0.91) (Figure 4).

Among the 72 CFI/LVEF measurements, 14 pertained to 9 patients with a right ventricular dysfunction. Reasons for the cardiogenic shock associated myocarditis and acute myocardial ischemia with right ventricular impairment, chemotherapy toxicity, and end-stage chronic heart failure. In these patients, CI and CFI were not correlated with LVEF (resp., *P* = 0.28; *P* = 0.34) (Table 5).

## 4. Discussion

The present study shows that CFI, obtained by the transpulmonary thermodilution function, is a reliable indicator of left ventricular ejection fraction in cardiogenic shock. Indeed, CFI was found to be statistically correlated with LVEF and to increase with inotropic infusion while not altered by fluid expansion. Moreover, a CFI <3.47 allowed predicting a LVEF ≤35% with good sensibility (81.1%) and specificity (63%).

Assessing LVEF in critically ill patients admitted to the ICU is a key aspect of their hemodynamic management, since detecting a low LVEF may lead to specific therapy such as inotropic infusion. Echocardiography is the gold standard for LVEF estimation: it is a reliable, safe, and noninvasive technique; however, echocardiography requires specific equipment and a competent operator 24/24 h, which is currently not possible in all ICUs. Thus, by providing two cardiac function indices, the PiCCO system could provide an interesting alternative to echocardiography in the assessment of LVEF. Our work confirmed the results of the two previous studies regarding the validity of CFI as an indicator of LVEF in critically ill ICU patients: Combes et al. [14] first demonstrated in 30 patients that CFI was correlated with LVEF assessed by transesophageal echocardiography (*r* = 0.87; *P* < 0.0001). A similar correlation was also described in 2009 by Jabot et al. [15] from 96 CFI/LVEF measurements (*r* = 0.67; *P* < 0.0001) involving 39 patients in which LVEF was obtained by transparietal echocardiography. In these studies, however, few patients presented severe cardiac dysfunction, as only 15% of the population was admitted for cardiogenic shock. Our study is the only study to assess CFI as a marker of LVEF in patients with severe cardiac impairment, as witnessed by a mean LVEF of 31%. Interestingly, the correlation between CFI and LVEF was slightly lower in our study than in the two previous studies (*r* = 0.52 versus 0.87 and 0.67, resp.). Because CFI represents the ratio between CO and GEDV, a severe dilatation of cardiac cavity such as that observed in chronic atrial fibrillations may underestimate CFI (and thus LVEF estimated with CFI). Combes et al. [14] excluded patients with nonsinus rhythm or with a known abdominal aortic aneurysm, whereas we did not exclude patients with a past history of arrhythmia or aneurysm. Moreover, in our population, we noted 31.5% chronic atrial fibrillation, which may explain the lower correlation between CFI and LVEF. Thus, CFI is correlated with LEVF even if the patient has a low LVEF or a past history of nonsinus rhythm. These patients are furthermore likely representative of the general ICU patient population.

In addition, CFI may also track the changes in LVEF under inotropic agent. Indeed, as previously demonstrated by Jabot et al. [15], we show herein that CFI statistically improved with inotropic treatment, whereas it was not altered by fluid infusion. The fact that only 4 fluid expansions were studied could be considered as a limitation of our study; however, it nevertheless confirms that these patients are representative of a population with cardiogenic shock, for example, with adequate intravascular volume.

A serious weakness of CFI is probably the existence of a right ventricular (RV) impairment. Since CFI represents the ratio between CO and GEDV, it reflects global myocardial contraction, in other words, left and right ventricular function [7, 23]. Hence, an isolated or preponderant RV impairment might underestimate CFI and consequently LVEF. In our series, CFI and LVEF in patients with severe RV impairment were not correlated, whereas Combes et al. excluded three patients with RV failure: for these patients, PiCCO clearly underestimated LVEF (more than 20% difference with the true LVEF) [14]. On the other hand, Jabot et al. [15] did not exclude patients with significant right heart failure: in their subgroup, CFI and LVEF were statistically correlated (*r* = 0.46); while the observed correlation coefficient was lower than the correlation for the whole population (*r* = 0.69), the difference between the two values was not statistically significant. In the present study, when considering the patients without a severe RV dysfunction (*n* = 58), CFI and LVEF are positively correlated (*r* = 0.55); this correlation coefficient is closed to the rate for the whole studied population (*r* = 0.52).

Considering that RV failure may underestimate LVEF or lead to a false negative, the PiCCO system cannot surrogate echocardiography for the assessment of LVEF. In clinical practice, a low CFI should alert the clinician to an impairment in systolic function (left and/or right): an echocardiography must therefore be performed to discriminate between right or left ventricular dysfunction; echocardiography can also provide critical information regarding diagnosis such as segmental wall-motion or valve abnormalities.

In conclusion, this study demonstrates that CFI is significantly correlated to LVEF in cardiogenic shock provided that patient does not present severe isolated right ventricular dysfunction. The PiCCO system is a simple and easily reproducible technique, which provides a consistent estimation of LVEF. However, it does not replace echocardiography: a low CFI should alert the physician to a possible impairment of LV systolic function, and an echocardiography must be performed to exclude right ventricular impairment. Once the LV dysfunction is confirmed, CFI allows a consistent monitoring of LV function under inotropic treatment.

## Figures and Tables

**Table 1:** Patient characteristics (*n* = 35).

*n* = 35		
Sex ratio	1.2	
Men (*n*; %)	19	(54.3%)
Age (years; SD)	66	±16
Underlying cardiovascular disease:		
(i) Preexisting cardiomyopathy (*n*; %)	21	(60%)
LVEF (%; SD)	39.7	±14
(ii) Chronic atrial fibrillation (*n*; %)	11	(31.5%)
SAPSII (*n*; SD)	54	±21
Causes of cardiogenic shock: (*n*; %)		
(i) Acute myocardial infarction	15	(42.9%)
(ii) End-stage cardiomyopathy	6	(17.1%)
(iii) Treatment toxicity (chemotherapy)	5	(14.3%)
(iv) Myocarditis	2	(5.7%)
(v) Complex heart rhythm disorder	2	(5.7%)
(vi) Thyrotoxicosis	1	(2.9%)
(vii) Unknown	4	(11.4%)
Need for life support techniques in ICU: (*n*; %)		
(i) Renal replacement therapy	12	(34.3%)
(ii) Mechanical ventilation	22	(62.8%)
(iii) Norepinephrine use	25	(71%)
(iv) Epinephrine use	1	(2.8%)
(v) Dobutamine use	35	(100%)
Mortality in the ICU (*n*; %)	19	(54%)

LVEF: left ventricular ejection function; SAPSII: simplified acute physiology score II; ICU: intensive care unit.

**Table 2:** Hemodynamic characteristics of pairs of CFI/LVEF measurements (*n* = 72).

	Mean	SD	
Patient data			
Norepinephrine (*μ*g/kg/min)	0.72	±0.79	*n* = 38
Epinephrine (*μ*g/kg/min)	0.77	—	*n* = 1
Dobutamine (*μ*g/kg/min)	9.4	±4.8	*n* = 63
Systolic arterial pressure (mmHg)	121	±20	*n* = 72
Diastolic arterial pressure (mmHg)	60	±12	*n* = 72
Mean arterial pressure (mmHg)	80	±15	*n* = 72
Heart rate (/min)	100	±19	*n* = 72
Echocardiography data			
Left ventricular ejection fraction (%)	31%	±11.7	*n* = 72
Pulmonary systolic arterial pressure (mmHg)	44	±12.3	*n* = 38
TAPSE (mm)	18.4	±4.7	*n* = 63
Transpulmonary thermodilution data			
Cardiac index (L/min/m^2^)	2.6	±0.8	*n* = 72
Cardiac function index (/min)	3	±1	*n* = 72
Global ejection fraction (%)	14.2	±6	*n* = 72
Global end diastolic volume (mL/m^2^)	820	±190	*n* = 72

CFI: cardiac function index; LVEF: left ventricular ejection function; TAPSE: tricuspid annular plane systolic excursion.

**Table 3:** Evolution of hemodynamic parameters before and after increasing dobutamine infusion; *n* = 18.

*n* = 18	Before		After		
	Mean	SD	Mean	SD	
Systolic arterial pressure (mmHg)	115	±19	117	±17.7	—
Diastolic arterial pressure (mmHg)	57	±11	55	±8.4	—
Mean arterial pressure (mmHg)	76	±13	75	±10	—
Heart rate/(min)	101	±25	105	±23	*P* = 0.078
Cardiac index (L/min/m^2^)	2.21	±0.55	2.95	±0.92	*P* = 0.0008
Cardiac function index (/min)	2.75	±0.87	3.28	±1.26	*P* = 0.0046
Global ejection function (%)	11.4	±3.9	13.6	±4.5	*P* = 0.0074
Global end-diastolic volume (L/m^2^)	788	±185	879	±255	*P* = 0.22
Dobutamine (*μ*g/kg/min)	5.3	±4.7	10.1	±5.7	—
Left ventricular ejection fraction % (*n* = 11)	27	±9	30.2	±9.4	—

**Table 4:** Measurements before and immediately after fluid infusion; *n* = 4.

*n* = 4	Before		After		
	Mean	SD	Mean	SD	
Systolic arterial pressure (mmHg)	124	±22	125	±24	—
Diastolic arterial pressure (mmHg)	65	±13	60	±18	—
Mean arterial pressure (mmHg)	83	±13	82	±17	—
Heart rate (/min)	95	±6	95	±6.2	—
Cardiac index (L/min/m^2^)	2.54	±1.18	2.45	±1.19	*P* = 0.62
Cardiac function index (/min)	4.4	±3	4.25	±2.9	*P* = 0.62
Global ejection function (%)	18.8	±11	19	±11	*P* = 0.85
Global end-diastolic volume (L/m^2^)	570	±140	610	±117	*P* = 0.62

**Table 5:** Patients with a right ventricular dysfunction; 14 measurements, 9 patients.

*n* = 14	Mean	SD
Echocardiography data		
Left ventricular ejection fraction (%)	29.9%	±9.8
Pulmonary systolic arterial pressure (mmHg)	47	±6.8
TAPSE (mm)	12.9	±1.5
Thermodilution		
Cardiac index (L/min/m^2^)	3	±1.1
Cardiac function index (/min)	2.95	±0.8
Global ejection function (%)	12	±3.7

TAPSE: tricuspid annular plane systolic excursion.
